# Non-Invasive Ventilation in Patients with Heart Failure: A Systematic Review and Meta-Analysis

**DOI:** 10.5935/abc.20170001

**Published:** 2017-02

**Authors:** Hugo Souza Bittencourt, Helena França Correia dos Reis, Melissa Santos Lima, Mansueto Gomes Neto

**Affiliations:** 1 Programa de Pós Graduação em Medicina e Saúde, Departamento de Fisioterapia - Curso de Fisioterapia da Universidade Federal da Bahia, Bahia, BA - Brazil; 2 Departamento de Fisioterapia - Curso de Fisioterapia da Universidade Federal da Bahia, Bahia, BA - Brazil

**Keywords:** Heart Failure, Noninvasive Ventilation, Exercise Tolerance, Review, Meta-Analysis

## Abstract

Non-invasive ventilation (NIV) may perfect respiratory and cardiac performance in patients with heart failure (HF).

The objective of the study to establish, through systematic review and meta-analysis, NIV influence on functional capacity of HF patients.

A systematic review with meta-analysis of randomized studies was carried out through research of databases of Cochrane Library, SciELO, Pubmed and PEDro, using the key-words: heart failure, non-invasive ventilation, exercise tolerance; and the free terms: bi-level positive airway pressure (BIPAP), continuous positive airway pressure (CPAP), and functional capacity (terms were searched for in English and Portuguese) using the Boolean operators AND and OR. Methodological quality was ensured through PEDro scale. Weighted averages and a 95% confidence interval (CI) were calculated. The meta-analysis was done thorugh the software Review Manager, version 5.3 (Cochrane Collaboration).

Four randomized clinical trials were included. Individual studies suggest NIV improved functional capacity. NIV resulted in improvement in the distance of the six-minute walk test (6MWT) (68.7m 95%CI: 52.6 to 84.9) in comparison to the control group.

We conclude that the NIV is an intervention that promotes important effects in the improvement of functional capacity of HF patients. However, there is a gap in literature on which are the most adequate parameters for the application of this technique.

## Introduction

HF is a clinical syndrome in which the heart has difficulty pumping blood, generating functional limitation with important cardiovascular, hemodynamic and metabolic alterations.^1-3^ HF patients have reduced FC, which may limit their performance of daily life activities (DLA) and reduce quality of life (QL).^4-6^ These alterations contribute to the increase of symptoms and to exercise intolerance, progressively reducing FC.^7^

Cardiac rehabilitation programs are being more and more recommended for this population, with the objective of minimizing the consequences of HF and improving the patient's QL. Cardiac rehabilitation is defined as a non-pharmacological treatment with an emphasis on the practice of physical exercise.^8^

Currently, some resources used in physical therapy are complementing a cardiac rehabilitation program for patients who initially cannot tolerate exercising. NIV with administration of CPAP is one of the utilized techniques. NIV may improve cardiac and respiratory performances of HF patients, considering it enhances oxygenation and pulmonary mechanics, so it can also improve FC.^7^

Traditionally, NIV has been used in respiratory insufficiency situations and in HF patients with the objective of reversing pulmonary edema and respiratory failure situations. The use of NIV and its effect on exercise tolerance have only recently started to be investigated, but there are controversies surrounding its efficacy and use in clinical practice. Systematic review with meta-analysis can solve conflict issues of individual studies and provide more reliable estimates of the efficacy of NIV use in HF patients. The aim of this work was to carry out a systematic review with meta-analysis about the use of NIV to improve FC in HF patients.

## Methods

A systematic review was realized, observing the criteria established by the Preferred Reporting Items for Systematic Reviews and Meta-Analyses (PRISMA) guideline.^9^

### Eligibility criteria

We included random clinical trials (RCT) that tested the use of NIV in patients over 18 years old, of both genders, with HF and without associated restrictive or obstructive pulmonary disease.

Evaluation measurements were: tolerance to effort; duration of exercise; perceived exertion; spirometry; lactatemia.

### Data source and research

Article research was done with databases from PubMed, *Cochrane Library*, SciELO and Physiotherapy Evidence Database (PEDro). In this research we included original articles published in English, Spanish and Portuguese up to August of 2015.

The initial search strategy consisted of four key-words (study design, participants, interventions, and result measurements). The utilized key-words were described from search terms Medical Subject Headings (MeSH) and Health Science Descriptors (DeCS) where, for the study design, we included: randomized clinical trial and controlled study. The group of participants used words referent to the disease such as HF, cardiac dysfunction or ventricular dysfunction. The key-words that were used for intervention were: NIV and exercise tolerance. The terms used for result measurements were: 6MWT, ergometry, ergospirometry, spirometry.

An experienced reviewer carried out the search and initial selection to identify the titles and abstracts of potentially relevant studies. Each abstract was assessed independently by two reviewers. If at least one of the reviewers considered a reference to be eligible, the article was obtained in its entirety. Both reviewers would then independently analyse the articles to select the ones to be included in the review. When there was a disagreement, the decision was made by the authors' consensus. A manual tracking of citations of the selected articles was also performed.

### Methodological quality assessment of the studies

The quality of the studies was assessed using the PEDro scale - the most widely used in the area of rehabilitation. This scale is based on the Delphi list,^10^ in order to measure the internal validity through the presence or absence of methodological criteria.^11^ The PEDro scale is made up of the following criteria: 1) specification of inclusion criteria (non-scored item); 2) random allocation; 3) confidential allocation; 4) group similarities in the initial or basal phase; 5) masking of subjects; 6) masking of the therapist; 7) masking of the assessor; 8) measurement of at least one primary outcome in at least 85% of the allocated subjects; 9) analysis of intention to treat; 10) comparison between groups of at least one primary outcome; 11) reports of variability measurements and parameter estimates of at least one primary variable. For each defined criterion in the scale, one point (1) is attributed to the presence of evidence quality indicator, and zero (0) is attributed to the absence of these indicators.^11^

### Statistical evaluation

Meta-analysis was done due to the similarity between studies in regards to the chosen intervention, patients' characteristics, and the variable distance covered in the 6MWT. Combined effect estimates were expressed as the mean difference between the groups. Statistical heterogeneity among the studies was assessed with Cochrane's Q test, and inconsistence test I^2^, in which values above 25% and 50% were considered indicative of moderate and high heterogeneity, respectively. Calculations were done using a fixed effect model, due to the low heterogeneity. An α value of 0.05 was considered significant. Analysis was done using the Review Manager, version 5.3 (*Cochrane Collaboration*).

## Results

**Figure 1:**
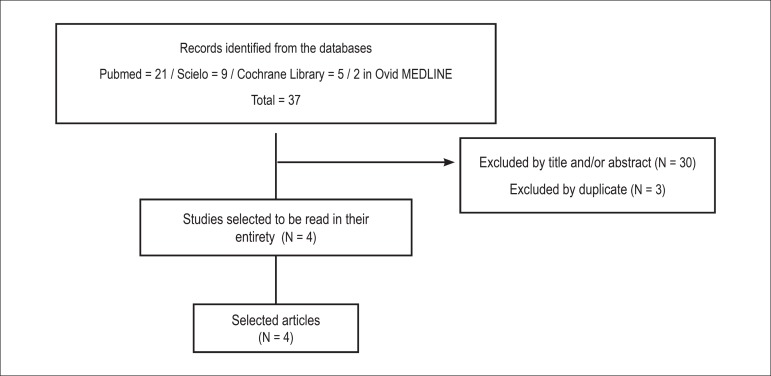
Flowchart of the article selection process.

We initially identified a total of 37 articles in the selected database research, 21 at PubMed, nine at SciELO, and seven at the Cochrane Library. After careful examination, 30 articles were excluded by title and/or abstract, and three by duplicate. The four remaining articles met the inclusion criteria and were selected, in their entirety, for reading (Figure 1).

### Study methodological quality analysis

Methodological quality analysis of the studies that met the inclusion criteria was done by two researchers in an independent way, in which a mean value of 6.2 was found using the PEDro scale (Table 1).

**Table 1:** Quality assessment of studies through PEDro scale

	O’Donnell et al.^12^	Wittmer et al.^14^	Chermont et al.^13^	Lima et al.^7^
1		✓		✓
2	✓	✓	✓	✓
3				✓
4		✓		✓
5				
6				
7		✓	✓	
8	✓	✓	✓	✓
9				
10	✓	✓	✓	✓
11	✓	✓	✓	✓
Total:	4	6	5	6
				**Mean: 6,2**

### Study characteristics

The four studies evaluated the impact of NIV in exercise tolerance of HF patients. Participants from all selected studies suffered from HF.^7,12-14^ The period of study publication was from 1999 to 2011, and the studied population size varied between 12^7,12,13^ and 22^14^ patients, amounting to 58 studied individuals. Mean age of participants varied between 46^7^ and 61^12^ years of age. The four studies were done with a population made up of both genders. ^7,12-14^ In three of the studies, participants belonged to functional class II to III according to the NYHA,^7,13,14^ and in one study, functional class varied between II to IV.^12^

Three of the studies used the 6MWT as an indicator of functional capacity of HF individuals,^7,13,14^ and only one study used the exercise test on a cycle ergometer^12^ as an evaluation tool.

### Non-invasive ventilation support characteristics

As an interface for NIV application, two studies^13,14^ chose the nasal mask, one study^7^ used the facial mask, and another opted for the oral mask.^12^

Three of the studies opted for CPAP,^7,13,14^ and one of them used CPAP and support pressure (SP).^12^ Wittmer et al.^14^ used CPAP of 8 cmH_2_O; Chermont et al.^13^ used a pressure of 3 cmH_2_O for 10 minutes in the CPAP group, progressing to 4 to 6 cmH_2_O, while in the placebo group, a pressure of 0 to 1 cmH_2_O was fixed. Lima et al.^7^ applied a pressure of 10 cmH_2_O, whereas O'Donnell et al.^12^ used CPAP and SP with positive end-expiratory pressure (PEEP) of 4.8 cm of H_2_O, comparing it to a control that used only 1 cmH_2_O.

The four selected articles that took part in the review used a control group and assessed the use of ventilator support in FC of HF patients.^7,12-14^ Two of them used CPAP in one single session before the exercises;^7,13^ one article used CPAP for 14 days;^14^ and only one article used both ventilatory support models (CPAP and SP) during the exercise.^12^

Characteristics of the included RCTs and of the intervention are described in Tables 2 and 3.

**Table 2:** Characteristics of studies included in the review

Study	Participants	Evaluation	Methods of evaluation			Results		
			Duration of exercise	Aerobic capacity	Dyspnea	Duration of exercise	Aerobic capacity	Dyspnea
O’Donnell et al.^12^	12 patients Men (11) Women (1) LVEF 35% FC/II/IV NYHA	Duration of exercise dyspnea	Time in minutes	No	BORG Scale	10.1±1.5 PS p < 0.01 8.7±1.1 CPAP p = 0.08 7.2 ± 1.0 control	No	p > 0.005
Wittmer et al. ^14^	22 patients Men (12) Women (10) LVEF 45% FC/II/III NYHA	Aerobic capacity	No	6MWT	No	No	↑6MWT (p<0.05)	No
Chermont et al.^13^	12 patients Men (8) Women (4) LVEF 45% FC/II/III NYHA	Aerobic capacity	No	6MWT	No	No	↑6MWT (p<0.05)	No
Lima et al.^7^	12 patients Men (8) Women (4) LVEF 35% FCII/III NYHA	Aerobic capacity	No	6MWT	BORG Scale	No	↑6MWT (p<0.05)	P: 0.009

6MWT: six-minute walk test; SP: support pressure; CPAP: continuous positive airway pressure; NYHA: New York Heart Association.

**Table 3:** Characteristics of interventions of studies included in the review

Study	Intervention time	Application time	Respiratory exercises	PEEP	NIVS (CPAP) Before exercise	NIVS (CPAP/SP) During exercise
O’Donnell et al.^12^	Session (1)	During exercise	No	PS/CPAP 4.8 cmH_2_O Control 1 cmH_2_O	No	Yes
Wittmer et al.^14^	Session (14)	30 minutes	3 x 10 repetitions SE/ DB/IH	CPAP 8 cmH_2_O	Yes	No
Chermont et al.^13^	Session (1)	30 minutes	No	CPAP 4-6 cmH_2_O Control 0-1 cmH_2_O	Yes	No
Lima et al.^7^	Session (1)	30 minutes	No	CPAP 10 cmH_2_O	Yes	No

NIVS: non-invasive ventilatory support; PEEP: positive end-expiratory pressure; SP: support pressure; CPAP: continuous positive airway pressure; SE: short expiration; DB: deep breath; IH: inspiratory hiccups.

### NIV effects on FC and pulmonary function

All works evaluated NIV impact on FC of HF patients, and all studies found, after the use of NIV, ^7,12-14^ an increase in exercise tolerance.

Chermont et al.^13^ observed an increase in tolerance to physical exercise with a longer distance covered in the 6MWT in HF patients, when they were submitted to 30 minutes of CPAP at 6 cmH_2_O before the test.

These results confirmed the findings of Wittmer et al.^14^ in their prospective randomized blind clinical trial, in which 22 patients (12 men and 10 women) were randomly divided to do 30 minutes of treatment with CPAP, respiratory exercises, and walking exercises (CPAP group), or respiratory exercise and walking exercise (control group) for 14 days. Through the 6MWT evaluation, one day before the treatment (day 0) and on the 4^th^, 9^th^, and 14^th^ days of treatment, the authors observed that patients in the CPAP group showed progressive improvement in the distance covered during 6MWT, reaching approximately 28% of base values at the end of the treatment, whereas the control group showed no significant changes. Despite having found an improvement in the distance covered, the authors were not able to determine with certainty if this positive outcome was due to an improvement on pulmonary function or to hemodynamic alterations.

In the study by O'Donnell et al.,^12^ the total time of exercise increased significantly during exercise with SP (p = 0.004), but only slightly with the use of CPAP (p = 0.079) in comparison to the control.

According to Lima et al.,^7^ there was significant improvement in the distance covered during 6MWT after the use of CPAP in only one application session of 30 minutes.

Of the four studies we found, three evaluated pulmonary function.^12-14^ In the study by O'Donnell et al.^12^ basal parameters of pulmonary function were within normal limits, except for a small reduction in forced vital capacity (FVC) and a reduction of the expiratory reserve volume (ERV). However, during exercise, patients presented significant increases in the end-expiratory lung volume. Even though the study by Chermont et al.^13^ cites that the patients were submitted to pulmonary function tests, it did not describe the results. Wittmer et al.^14^ observed an increase in FVC in patients treated with CPAP, reaching a maximum value or 16% of the basal value on the 9^th^ day of treatment, in comparison to the control group. In the same way, FEV1 values increased progressively, reaching a maximum value of 14% on the 14^th^ day of treatment.

### NIV effects on lactate concentration

Only the study by Lima et al.^7^ evaluated the lactate concentration in HF patients after the 6MWT with previous application of CPAP. The patients who were submitted to NIV obtained a lower lactate concentration at the end of the test in comparison to controls.

### NIV effects on the duration of exercise

Patients who used CPAP before the 6MWT walked a longer distance in meters than those in the control group.^7,13^ The use of CPAP, added to the respiratory exercises and walking exercises, also induced a significant increase in the covered distance during the 6MWT in comparison to a control group that did only respiratory and walking exercises.^14^ The use of SP in association to exercise on a cycle ergometer, in comparison to CPAP and placebo, was more effective in the evaluation of permanence time in the exercise on a cycle ergometer, and in the evaluation of subjective perceived exertion through the BORG scale.^12^ There were also differences in the use of CPAP in comparison to the placebo. CPAP mode increased the duration of the exercise, with a smaller effort rate by the BORG scale.

**Figure 2:**
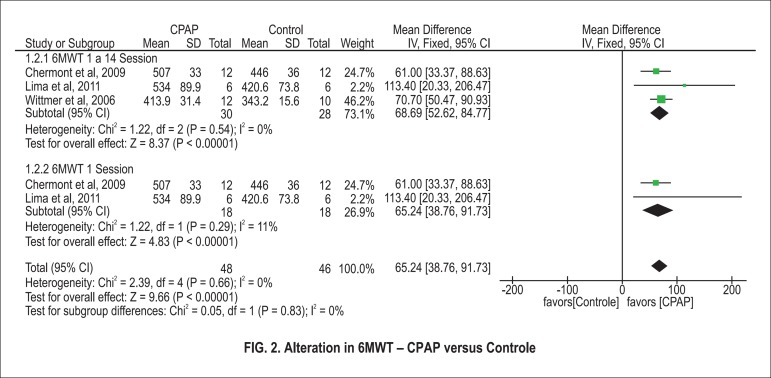
CPAP versus Control: 6MWT. Review Manager (RevMan). Version 5.2 The Cochrane Collaboration, 2013.

Three studies evaluated the 6MWT. Of these, two evaluated the effect of one NIV session, while the third evaluated the effect of 14 sessions of NIV. The meta-analysis of the three showed (Figure 2) a significant difference in the 6MWT distance (68.7 m 95% CI: 52.6 to 84.9; N=58) for participants of the NIV group compared to controls. When combining only the two studies that used NIV in one single session, meta-analysis showed (Figure 2) a significant difference in the 6MWT distance (65.2 m 95% CI: 38.8 a 91.7; N=36) for participants in the NIV group in comparison to controls.

## Discussion

This systematic review had the objective of identifying the scientific evidence on the impact of NIV on HF patients' FC. The results indicate a significant improvement on tolerance to exercise in HF patients after NIV intervention, in comparison to the control group.

NIV is being used as an important tool in the treatment of HF patients for the improvement of ventilatory efficiency during exercise.^15,16^ This fact may be associated to factors such as improvement in oxygenation, attenuation of the metaboreflex, improvement in the ventilation/perfusion ratio (V/Q), airway patency and consequent reduction of ventilatory work and fatigue.^7,12,17^

HF patients present decreased tolerance to effort associated to an increase of dyspnea and muscle fatigue.^18^ Previous use of CPAP increased the distance patients covered during the 6MWT and prolonged duration of exercise on the cycle ergometer when used simultaneously with exercises.^7,12-14^

Evaluation of HF patients is extremely relevant; thus, cardiopulmonary exercise testing (CPET) is the reference standard and the most specific test for ventilatory evaluation during physical exercise - it not only measures FC, which is directly linked to the severity of HF, it can also evaluate the patient's oxygen consumption (VO2). However, since the cardiopulmonary test is complex and costly, the 6MWT proves to be an efficacious tool in FC evaluation.^19^

The 6MWT can be related to daily physical activities, and so it is a submaximal test of simple execution and low cost. The 6MWT is an excellent option, able to assess FC, and working as a predictor of mortality in this population.^17-19^ Moreover, studies report that the distance covered in the test is associated to the functional classification of the NYHA.^20-24^

The 6MWT has been used in three studies to evaluate the distance covered by the patients.^7,13,14^ Lima et al.^7^ found significant differences in the distance covered during the 6MWT in patients submitted to NIV with CPAP, in comparison to the control group. The study's results corroborate the work done by Chermont et al.^13^ in which NIV promoted an increase in the distance covered (NIV: 507 m; placebo: 446 m; p = 0.001) by patients with increased tolerance to exercise.

Previous CPAP administration in HF decreases respiratory discomfort in patients, generating lower cardiac work during exercise.^12,25^ Smaller quantities of lactate have also been attributed to the use of CPAP in patients after the 6MWT.^7^

NIV is an important instrument used to perfect the treatment of patients, with significant improvement in the performance of physical activities.^26^ Pulmonary function may be decreased in HF, having a direct relationship with the reduction in FC and in the performance of DLAs.^27,28^ Wittmer et al.^14^ have demonstrated that treatment with CPAP progressively increased FVC and FEV1 in HF patients when compared to the control group. This improvement may have occurred due to the increase in functional residual capacity and opening of collapsed alveoli.^7,12-14^

In the study by Wittimer et al.^14^ a clinical implication in relation to FVC was observed as a component of the outcome associated to FC after repercussion of NIV application. The CPAP group showed progressive increase of FVC, reaching a maximum of 16% of the basal value on the 9^th^ day of treatment, with no additional improvement on the 14^th^ day of treatment. VEF1 values increased progressively and reached a maximum of 14% on the 14^th^ day of treatment with CPAP, with no significant changes in the control group. The authors concluded that the treatment with CPAP, for two weeks, increased pulmonary function of HF patients, consequently improving tolerance to activities.

The increase in respiratory work in HF is associated to a decreased diaphragm perfusion. Due to this event, patients who are decompensated by the disease evolve with muscle fatigue in lower limbs, caused by an increase in peripheral vascular resistance.^12,25^ Obtainment of lower resistance to airflow in the airways with administration of positive pressure,^7,12,19^ and reduction in respiratory discomfort or fatigue in lower limbs^7,12-14^ are factors that can also explain the improvement in FC with the use of NIV associated to exercise.

Patients who used non-invasive ventilatory support (NIVS) increased their FC when they used a PEEP superior to 4 cmH_2_O. Studies that compared the use of a lower value PEEP or placebo mode proved it to be inefficacious when compared to a higher level PEEP.^12,13,29^

Enlargement of the cardiac area generates a volume overload in cardiac cavities. NIV decreasesthis volume overload, momentarily, with an increase in cardiac contractility, which occurs with the advent of transmural pressure reduction.^7,30,31^ Moreover, NIV favors a pressure condition, which promotes an improvement in gas exchange by simple recruitment and stabilizes alveolar units.^32^

Despite the positive results, patient care and monitoring are necessary during NIV application. The decrease in cardiac debit and hypoperfusion seem to challenge the use of this technique. However, the positive intrathoracic pressure offered by NIV influences the patient's hemodynamic condition, with the decrease of cardiac preload and afterload due to the reduced transmural pressure.^33^

Tkacova et al.^34^ observed, after treatment with CPAP during three months, a significant decrease in atrial natriuretic peptide (ANP) in the plasma of HF patients. Patients submitted to CPAP had a decrease in pulse pressure correlated to an increase in the ejection fraction originated by the reduction of the transmural pressure.^35^

The presence of biases in these studies leads to conclusions that systematically tend not to be completely reliable.^36^ All selected studies presented high risk of biases in regards to allocation confidentiality, which is extremely important. O'Donnell et al.^12^ for instance, presented uncertain risk of masking, one of the factors that may significantly alter the study's result.

## Conclusion

This systematic review with meta-analysis showed that NIV is an effective method for the improvement of exercise tolerance in HF patients. However, there is a gap in literature regarding which are parameters that are the most adequate for the application of this technique, and that promote the best results in FC performance. Further research is necessary to determine a standardization regarding NIV application, so that this technique can contribute, more efficiently, to the treatment of HF patients.
